# Neddylation inhibition activates the protective autophagy through NF-κB-catalase-ATF3 Axis in human esophageal cancer cells

**DOI:** 10.1186/s12964-020-00576-z

**Published:** 2020-05-12

**Authors:** Yupei Liang, Yanyu Jiang, Xing Jin, Ping Chen, Yongqing Heng, Lili Cai, Wenjuan Zhang, Lihui Li, Lijun Jia

**Affiliations:** 1 Cancer Institute, Longhua Hospital, Shanghai University of Traditional Chinese Medicine, Shanghai, 200032 China; 2 School of Basic Medical Sciences, Zhengzhou University, Zhengzhou, 450001 China; 3 Department of Breast Surgery, Key Laboratory of Breast Cancer in Shanghai, Fudan University Shanghai Cancer Center, Shanghai, China

**Keywords:** Neddylation, MLN4924, NF-κB/catalase/ATF3, Autophagy, Apoptosis, Esophageal Cancer

## Abstract

**Background:**

Protein neddylation plays a tumor-promoting role in esophageal cancer. Our previous study demonstrated that neddylation inhibition induced the accumulation of ATF4 to promote apoptosis in esophageal cancer cells. However, it is completely unknown whether neddylation inhibition could induce autophagy in esophageal cancer cells and affect the expression of other members of ATF/CREB subfamily, such as ATF3.

**Methods:**

The expression of relevant proteins of NF-κB/Catalase/ATF3 pathway after neddylation inhibition was determined by immunoblotting analysis and downregulated by siRNA silencing for mechanistic studies. ROS generation upon MLN4924 treatment was determined by H2-DCFDA staining. The proliferation inhibition induced by MLN4924 was evaluated by ATPLite assay and apoptosis was evaluated by Annexin V /PI double staining.

**Results:**

For the first time, we reported that MLN4924, a specific inhibitor of Nedd8-activating enzyme, promoted the expression of ATF3 to induce autophagy in esophageal cancer. Mechanistically, MLN4924 inhibited the activity of CRLs and induced the accumulation of its substrate IκBα to block NF-κB activation and Catalase expression. As a result, MLN4924 activated ATF3-induced protective autophagy, thereby inhibiting MLN4924-induced apoptosis, which could be alleviated by ATF3 silencing.

**Conclusions:**

In our study, we elucidates a novel mechanism of NF-κB/Catalase/ATF3 pathway in MLN4924-induced protective autophagy in esophageal cancer cells, which provides a sound rationale and molecular basis for combinational anti-ESCC therapy with knockdown ATF3 and neddylation inhibitor (e.g. MLN4924).

Video abstract

**Graphical abstract:**

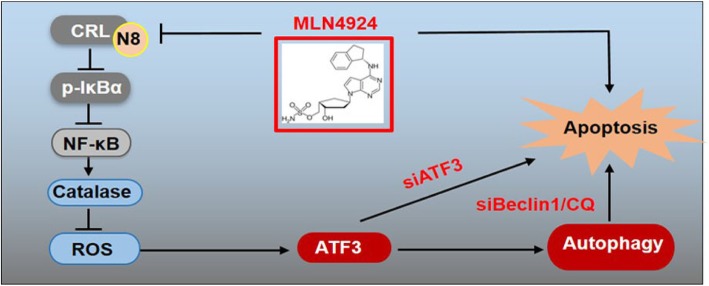

## Background

Post-translational modification of proteins plays crucial roles in the regulation of tumorigenesis and progression. Protein neddylation is an important post-translational modification that adds the ubiquitin-like molecule NEDD8 to substrate proteins [1]. In the process of neddylation, NEDD8 is first catalyzed by Nedd8-activating enzyme (NAE, NAE1 and UBA3 isodimer), transferred to NEDD8 conjugating enzyme E2, and then conjugated to substrates via a specific NEDD8-E3 ligase [2, 3]. The best-characterized substrates of neddylation pathway are the cullin subunits of Cullin-RING E3 ubiquitin ligase (CRL) [2]. As the largest family of E3 ubiquitin ligases, the activation of CRL requires the attachment of NEDD8 to cullin, then to promote ubiquitination and degradation of CRL substrates [4, 5]. Accumulated studies show that protein neddylation is elevated in multiple human cancers and inhibition of this pathway has been developed as a promising anticancer strategy [4, 6–10].

MLN4924, a specific inhibitor of NAE, was previously indentified through a high-throughput screening [5, 11, 12]. MLN4924 could block cullin neddylation to inhibit the activation of CRLs, thus inducing the accumulation of tumor-suppressive CRL substrates to inhibit tumor growth and metastasis both in vitro and in vivo [2, 4, 5, 13–19]. For its significant anticancer efficacy and well-tolerated toxicity, MLN4924 has been advanced into several phase II/III clinical trials against several solid tumors and hematologic malignancies [5, 9, 11, 20, 21]. Mechanistic studies showed that MLN4924 effectively induced DNA re-replication stress/DNA damage response, cell-cycle arrest, apoptosis or senescence in a cell-type-dependent manner [22–25]. Moreover, MLN4924 also induced pro-survival autophagic responses in cancer cells partially via modulating the HIF1-REDD1-TSC1 or Deptor-mTORC1 pathways [26–28].

Activating transcription factor 3 (ATF3) is a stress-responsive factor that belongs to the ATF/CREB subfamily of the basic region leucine zipper (bZIP) family [29]. ATF3 expression is often correlated with cellular damage and strongly induced by different stress signals. Functionally, ATF3 acts as pro-apoptotic or anti-apoptotic signals upon drug treatments in cell context-dependent manners [30–32]. Targeting neddylation has emerged as an attractive anticancer strategy, however, whether and how ATF3 response to neddylation-targeted therapy remains elusive. Here, for the first time, we reported that neddylation inhibition with MLN4924 induces the accumulation of ATF3 to trigger pro-survival autophagy by modulating NF-κB-Catalase-ROS-ATF3 axis in esophageal cancer cells, highlighting targeting ATF3-mediated autophagy as a potential strategy to enhance neddylation-targeted anti-ESCC therapy.

## Methods

### Cell lines, culture and reagents

Human ESCC cell lines EC1 and Kyse450 were cultured in Dulbecco’s Modifed Eagle’s Medium (Hyclone), containing 10% FBS (Biochrom AG) and 1% penicillin–streptomycin solution, at 37 °C with 5% CO_2_. Chloroquine (CQ), Bafilomycin A1 (BafA1) and 3-methyladenine (3MA) were purchased from Sigma. MLN4924 was synthesized and prepared as previously described.

### Cell viability

Cells were inoculated into 96 well plates (2 × 10^3^ cells per well) and treated with DMSO or MLN4924. According to the manufacturer’s protocol, cell proliferation was measured by ATPLite luminescence analysis kit (PerkinElmer).

### Immunoblotting

Cell lysates were prepared for immunoblotting (IB) analysis with antibodies against ATF3 (Cell Signaling Technology), LC3 (Cell Signaling Technology), cleaved PARP (Cell Signaling Technology), IκBα (Cell Signaling Technology), p-IκBα (Cell Signaling Technology), Catalase (Cell Signaling Technology), GAPDH (Cwbiotech) and ACTIN (Cwbiotech). Image J software was used for densitometric analysis.

### Gene silencing using siRNA

EC1 and Kyse450 cells were transfected with siRNA oligonucleotides, synthesized by Ribobio, using Lipofectamine 2000. The sequences of siRNA are as follows:

- siATF3: ATGTCCTCTGCGCTGGAAT;
- siIκBα: GCCAGAAATTGCTGAGGCA.

### Construction of plasmid

The pcDNA3-ATF3 plasmid is kindly provided by Prof. Dakang Xu (Ruijin Hospital, Shanghai Jiaotong University School of Medicine, Shanghai) [33]. Briefly, full-length complementary DNA (cDNA) of ATF3 was cloned into the pcDNA3 vector using standard protocols [33]. Full-length complementary DNA (cDNA) of Catalase was cloned into the p-CMV vector using standard protocols. All constructions were confirmed by DNA sequencing before further applications.

RNA isolation and quantitative polymerase chain reaction (Q-PCR).

Total RNA was extracted by Ultrapure RNA kit (Cwbiotech). RNA (1.0 μg) was purified and reversely transcribed by PrimeScript® RT Master (Takara) according to the manufacturer’s instructions. The cDNA was quantifed by real-time quantitative PCR using SYBR® Green Real-Time PCR Master Mixes (Applied Biosystems) and a 7500 Real-time PCR system (Applied Biosystems) according to the manufacturer’s instructions.

### Detection of apoptosis

Cells were treated with MLN4924 at a specified concentration for appointed time. Apoptosis was detected by Annexin V-FITC/PI apoptosis kit (Biovision, Inc).

### Quantifcation of reactive oxygen species

The quantification of reactive oxygen species(ROS) production was monitored by cell permeable ROS indicator 2′,7′- dichlorodihydrofuorescein diacetate (H2-DCFDA) (Sigma). The role of ROS in autophagy was evaluated by free radical scavenger NAC (Beyotime). Cells were pre-incubated with 50 μM NAC for 2 h, then co-incubated with the indicated chemicals and assessed for autophagy or ROS production as described above.

### Statistical analysis

GraphPad prism5 software was used to evaluate the statistical significance of the differences among groups. Unmatched 2-tailed t-test was used to compare the parameters between groups. The level of significance was set at *P* < 0.05.

## Results

### MLN4924 induced ATF3 transactivation

**Fig. 1:**
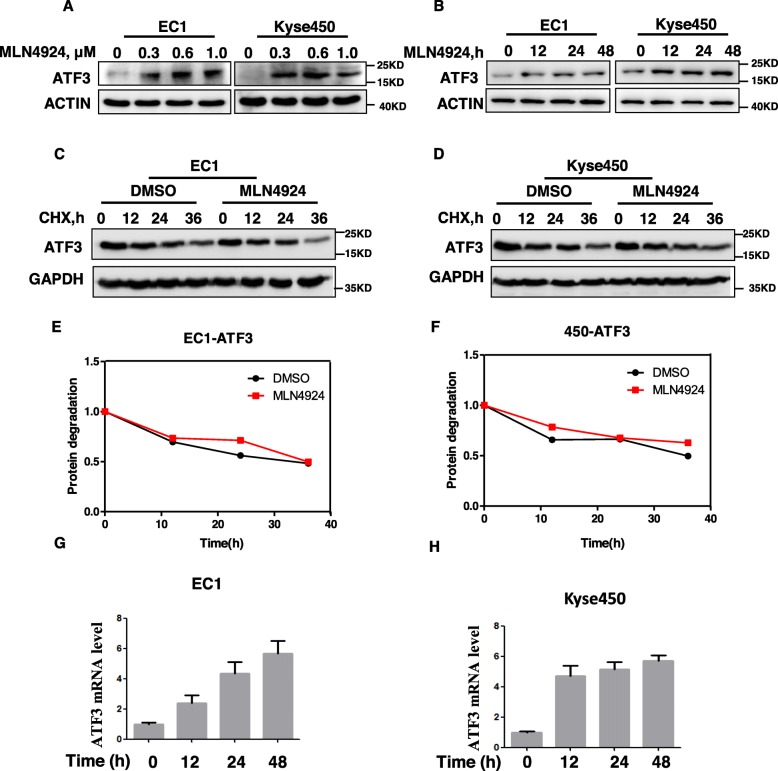
MLN4924 induced ATF3 accumulation at the transcriptional level. **a** EC1 and Kyse450 cells were treated with MLN4924 for 24 h and cell lysates were assessed by IB with specific antibody against ATF3. **b** EC1 and Kyse450 cells were treated with MLN4924 for 0 h, 12 h, 24 h or 48 h with 0.6 μmol/L MLN4924 and cell lysates were assessed by IB with specific antibody against ATF3. **c**-**f** MLN4924 did not enhance the protein stability of ATF3. EC1 and Kyse450 cells were pretreated with 0.6 μmol/L MLN4924 for 12 h to increase the basal protein level of ATF3. Cells were then washed with PBS to remove the residual of MLN4924 and divided into two groups, which were further treated with 50 μg/mL of cycloheximide in the presence or absence of MLN4924 (0.6 μmol/L) for indicated period of time and then collected for IB analysis. The protein level was quantified by densitometric analysis using Image J software. **g**-**h** MLN4924 increased ATF3 at the transcriptional level. EC1 and Kyse450 cells were treated with MLN4924 for 0 h, 12 h, 24 h or 48 h with 0.6 μmol/L MLN4924 and RNA were extracted and analyzed by qPCR (normalized to GAPDH)

Since MLN4924 treatment induced the accumulation of ATF4 [1], a member of ATF/CREB subfamily, in esophageal cancer cells, we reasoned whether neddylation inhibition could affect the expression of other members of ATF/CREB subfamily, such as ATF3. Therefore, we determined the effects of MLN4924 on the expression of ATF3 in EC1 and Kyse450 cells. We found that MLN4924 significantly induced the accumulation of ATF3 in both EC1 and Kyse450 cells (Fig. 1a and Fig. 1b). Given that ATF3 could be ubiquitinated by CRL/SCF ubiquitin ligase and degraded by proteasome [34], we further determined the stability of ATF3 using cycloheximide (CHX) chase assay upon MLN4924 treatment. Unexpectedly, the half-life of ATF3 was not influenced upon the inactivation of neddylation-CRL axis by MLN4924 (Fig. 1c-d). The expression level of ATF3 was further quantified by densitometric analysis using Image J software. GAPDH was used as the loading control (Fig. 1e-f). Thus, we further determined the expression of ATF3 using real-time PCR for mRNA quantification. As shown in Fig. 1g-h, the expression of ATF3 in transcriptional level was statistically elevated in EC1 and Kyse450 cells upon MLN4924 treatment. These findings collectively demonstrated that MLN4924 induced the transactivation of ATF3.

### ATF3 silencing blocked MLN4924-induced autophagy

**Fig. 2:**
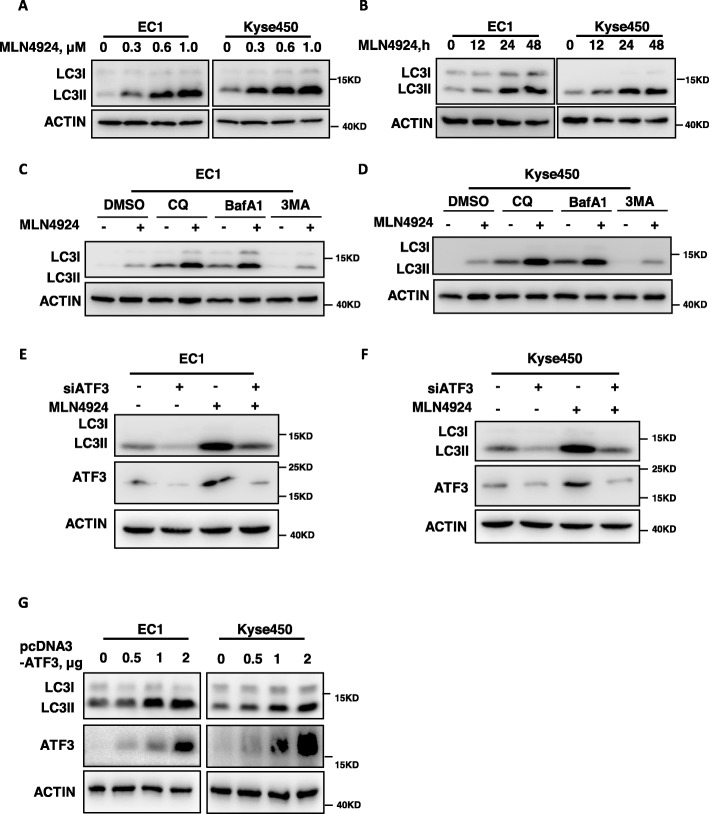
ATF3 silencing blocked MLN4924-induced autophagy. **a** Treatment of MLN4924 induced the conversion of LC3-I to LC3-II in a dose dependent manner in both EC1 and Kyse450 cells. Cells were treated with the indicated concentrations of MLN4924 for 48 h and cells were collected and subjected to IB analysis for the expression of LC3. Actin was used as an equal loading control. **b** MLN4924 induced the conversion of LC3-I to LC3-II in a time-dependent manner in both EC1 and Kyse450 cells. Cells were treated with the 0.6 μmol/L of MLN4924 for indicated time and then cells were collected and subjected to IB analysis for the expression of LC3. Actin was used as an equal loading control. **c**-**d** Autophagic flux analysis. EC1 and Kyse450 cells, treated with DMSO or MLN4924 for 48 h, were incubated with or without CQ (50 μM), BafA1 (50 nM) or 3MA (5 mM) for 8 h. The treated cells were then collected and subjected to IB analysis. Actin was used as an equal loading control. **e**-**f** ATF3 is required for MLN4924 induced autophagy in esophageal cancer cells. EC1 and Kyse450 cells were transfected with control or ATF3 siRNA for 48 h and then treated with 0.6 μmol/L MLN4924 for 48 h. ATF3 Knockdown efficiency and conversion of LC3-I to LC3-II were assessed by IB analysis. **g** ATF3 overexpression increase autophagy. EC1 and Kyse450 cells were transfected with pcDNA3-ATF3 (0 μg, 0.5 μg, 1 μg, 2 μg) for 48 h. ATF3 overexpression efficiency and conversion of LC3-I to LC3-II were assessed by IB analysis

We investigated whether and how ATF3 mediates MLN4924-induced cellular responses. As shown, MLN4924 induced the conversion of LC3-I to LC3-II, a classical marker of autophagy, in dose-dependent and time-dependent manner in EC1 and Kyse450 cells (Fig. 2a-b). In addition, we performed autophagic flux analysis by treating cells with classical autophagy inhibitors, including Chloroquine (CQ), bafilomycin A1 (BafA1) and 3-methyladenine (3MA), respectively. As expected, CQ and BafA1 enhanced, while 3MA inhibited the accumulation of LC3-II, indicating that autophagic flux was intact and supraphysiological autophagic response was induced by MLN4924 treatment (Fig. 2c and d). To determine the potential role of ATF3 in MLN4924-induced autophagy, the expression of ATF3 is down-regulated via siRNA silencing. As shown in Fig. 2e-f, the ATF3 knockdown completely blocked the conversion of LC3-I to LC3-II upon MLN4924 treatment, indicating a crucial role of ATF3 in MLN4924-induced autophagy in esophageal cancer cells. To further validate the role of ATF3 in increasing autophagy, ATF3 was ectopically expressed in EC1 and Kyse450 cells. Similar to ATF3 accumulation induced by MLN4924 treatment, ATF3 overexpression via transfecting the plasmid of pcDNA3-ATF3 also significantly induced the conversion of LC3-I to LC3-II in a dose-dependent manner in EC1 and Kyse450 cells (Fig. 2g). Taken together, these findings highlight a pivotal role of ATF3 expression in the regulation of autophagy.

### MLN4924-induced autophagy was a survival signal and ATF3 silencing enhanced drug toxicity via inducing apoptosis

**Fig. 3:**
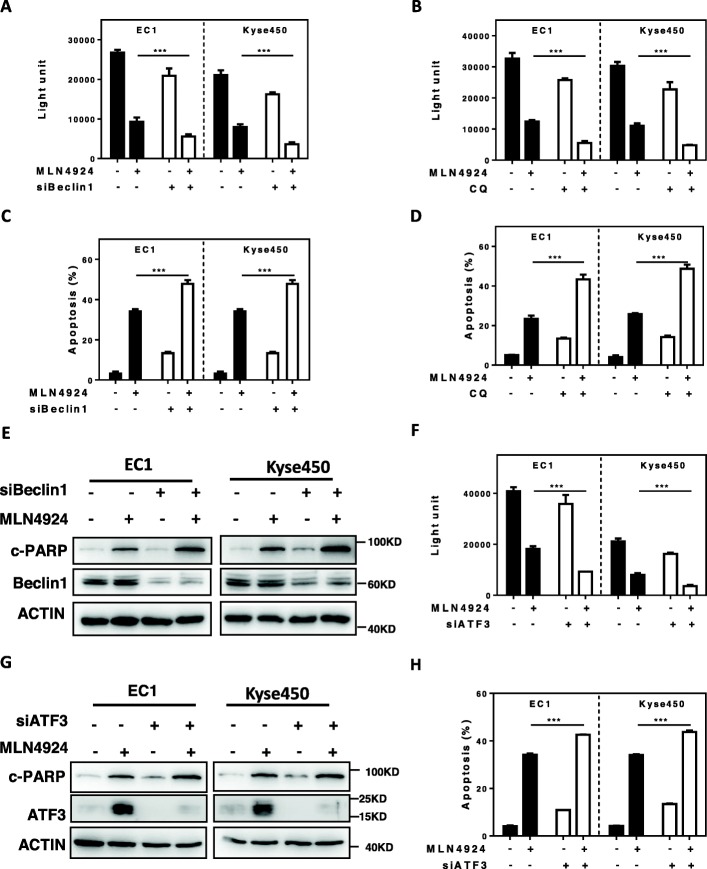
MLN4924-induced autophagy was a survival signal and ATF3 silencing enhanced drug toxicity via inducing apoptosis. **a**-**b** The proliferation inhibition by MLN4924 was significantly increased by simultaneously blocking autophagy with siBeclin1(**a**) or CQ(**b**). The combination of siBeclin1 or CQ with MLN4924 in EC1 and Kyse450 cells significantly increased proliferation inhibition by ATPLite assay. **c**-**d** Apoptosis induced by MLN4924 was significantly increased by simultaneously blocking autophagy with siBeclin1 or CQ. The combination of siBeclin1(**c**)or CQ (**d**) with MLN4924 in EC1 and Kyse450 cells significantly increased apoptosis by Annexin V /PI double staining. **e** Beclin1 knockdown increased cleaved PARP expression induced by MLN4924. EC1 and Kyse450 cells were transfected with control or Beclin1 siRNA for 48 h and then treated with 0.6 μmol/L MLN4924 for 48 h. Knockdown efficiency and cleaved PARP were assessed by IB analysis. **f**-**h** Blocking autophagy by siATF3 remarkably suppressed cell proliferation and induced cell apoptosis compared with MLN4924 alone. EC1 and Kyse450 cells were transfected with control or ATF3 siRNA for 48 h and then treated with 0.6 μmol/L MLN4924 for 48 h. Cell proliferation suppressed by MLN4924 was further significantly decreased by simultaneously silencing of ATF3 expression (**f**). Knockdown efficiency and cleaved PARP were assessed by IB analysis (**g**). Apoptosis detection by either annexin V and PI double staining (**h**)

Next we determined the role of ATF3-mediated autophagy response upon MLN4924 treatment in esophageal cancer cells. Firstly, we blocked autophagy via both pharmacological (siRNA silencing of autophagy essential gene Beclin1) and genetic approaches (using autophagy inhibitor CQ), and found that blocking autophagy significantly enhanced the MLN4924-induced proliferation inhibition in EC1 and Kyse450 cells (Fig. 3a-b), indicating the pro-survival role of MLN4924-induced autophagy. Consistently, the inhibition of autophagic response by siBeclin1 and CQ significantly enhanced MLN4924-induced apoptosis (Fig. 3c-d), as best evidenced by the increase of Annexin V-positive cell populations and the accumulation of cleaved PARP, a classical marker of apoptosis (Fig. 3e). Furthermore, we found that ATF3 knockdown significantly enhanced MLN4924-induced proliferation inhibition (Fig. 3f). In addition, ATF3 knockdown significantly enhanced MLN4924-induced apoptosis, as evidenced by the accumulation of cleaved PARP (Fig. 3g) and the increase of Annexin V-positive cell populations (Fig. 3h). These results demonstrated that MLN4924 induced the ATF3-mediated autophagy as a pro-survival signal in esophageal cancer cells.

### ATF3 accumulation is mediated by ROS upon MLN4924 treatment

**Fig. 4:**
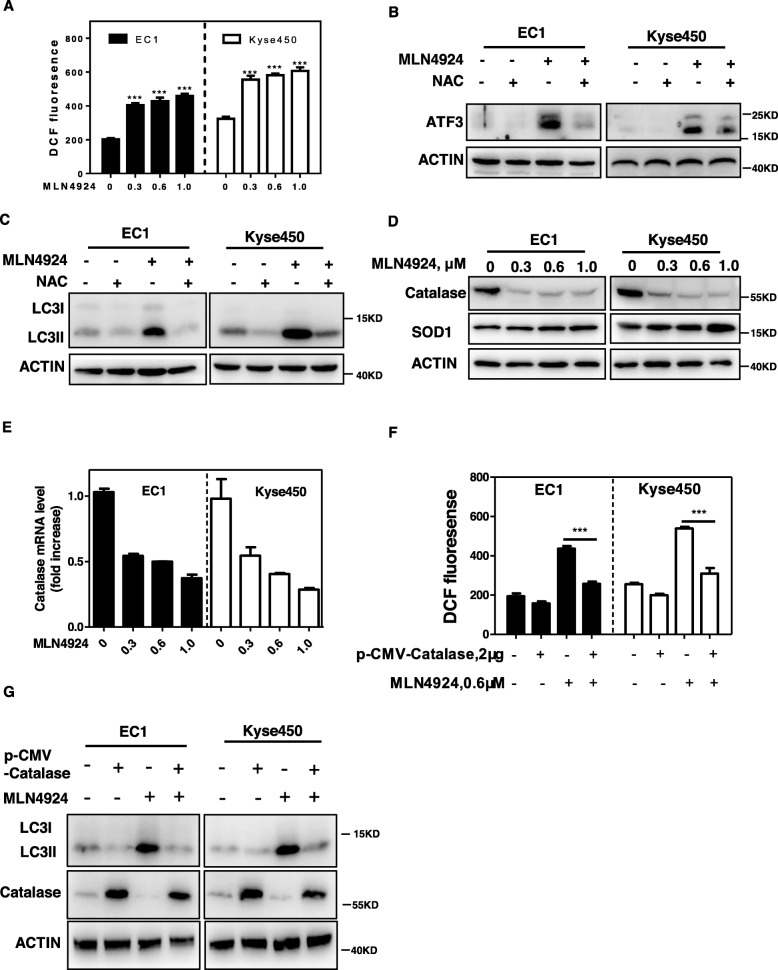
ATF3 accumulation is mediated by ROS upon MLN4924 treatment. **a** MLN4924 treatment induced ROS generation. EC1 and Kyse450 cells were treated with different dose of MLN4924 for 24 h and ROS generation was determined by H2-DCFDA staining. **b** NAC, a classical ROS scavenger, attenuated ATF3 expression. EC1 and Kyse450 cells were treated with 0.6 μmol/L MLN4924 alone or MLN4924 + NAC for 48 h and subjected to IB analysis for the expression of ATF3. Actin was used as an equal loading control. **c** Reduction of ROS by NAC significantly inhibited MLN4924-induced autophagy. EC1 and Kyse450 Cells were treated with 0.6 μmol/L of MLN4924 alone or MLN4924 + NAC for 48 h and subjected to IB analysis for the expression of LC3. Actin was used as an equal loading control. **d** MLN4924 decreased the expression of Catalase and did not affect the expression of SOD1. EC1 and Kyse450 Cells were treated with the indicated concentrations of MLN4924 for 48 h and subjected to IB analysis for the expression of Catalase, SOD1. Actin was used as an equal loading control. **e** MLN4924 decreased Catalase at the transcriptional level. EC1 and Kyse450 cells were treated with different dose of MLN4924 for 24 h and RNA were extracted and analyzed by qPCR (normalized to GAPDH). **f** Catalase overexpression attenuated MLN4924-induced ROS production. EC1 and Kyse450 cells were transfected with 2 μg p-CMV-Catalase for 24 h and then treated with 0.6 μmol/L MLN4924 for 24 h. ROS generation was determined by H2-DCFDA staining. **g** Catalase overexpression reversed MLN4924-induced autophagy. EC1 and Kyse450 cells were transfected with 2 μg p-CMV-Catalase for 24 h and then treated with 0.6 μmol/L MLN4924 for 24 h. Catalase overexpression efficiency and conversion of LC3-I to LC3-II were assessed by IB analysis

Previous studies indicated that ROS could induce ATF3 expression and MLN4924 could induce ROS production in cancer cells [35, 36]. Based on these findings, we determined whether MLN4924-induced ATF3 accumulation is mediated by ROS. We firstly detected cellular ROS level with the cell permeable ROS indicator, 2′, 7′-dichlorodihydrofuorescein diacetate (H2-DCFDA), and found that MLN4924 significantly induced ROS production in both EC1 and Kyse450 cells (Fig. 4a). Furthermore, we determined the role of ROS in MLN4924-induced ATF3 expression and autophagy. As shown in Fig. 4b and c, pre-treatment of esophageal cancer cells with NAC, a classical ROS scavenger, dramatically attenuated the expression of ATF3 (Fig. 4b) and inhibited the autophagic response (Fig. 4c) induced by MLN4924.

Given that Catalase and SOD1, as ubiquitous antioxidant enzymes, could inhibit the generation of ROS [37], we determine whether MLN4924 regulates the expression of Catalase or SOD1. As shown in Fig. 4d-e, MLN4924 significantly reduced the expression of Catalase at protein and mRNA levels but do not reduce the expression of SOD1. In order to determine the role of Catalase in MLN4924-induced ROS production and autophagy, Catalase was ectopically expressed in EC1 and Kyse450 cells with or without MLN4924 treatment. Indeed, Catalase expression significantly reversed MLN4924-induced ROS production (Fig. 4f). Moreover, Catalase expression completely blocked the conversion of LC3-I to LC3-II upon MLN4924 treatment (Fig. 4g). These findings demonstrated that MLN4924 induced ROS/ATF3 axis to trigger autophagy by reducing Catalase expression in esophageal cancer cells.

### MLN4924 activated autophagy by modulating NF-κB-catalase-ATF3 axis

**Fig. 5:**
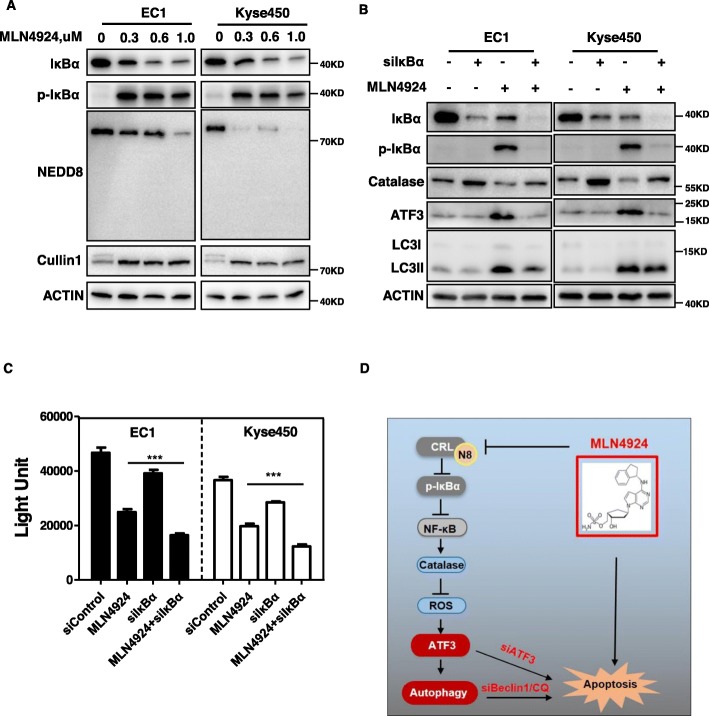
MLN4924 activated autophagy by modulating NF-κB-Catalase-ATF3 axis. **a** MLN4924 inhibited the activation of NF-κB pathway. EC1 and Kyse450 Cells were treated with the indicated concentrations of MLN4924 for 48 h and subjected to IB analysis for the expression of IκBα, p-IκBα, NEDD8. Actin was used as an equal loading control. **b** The effect of inhibition of NF-κB pathway caused by MLN4924 treatment was on both Catalase and ATF3 expression. EC1 and Kyse450 cells were transfected with control or siIκBα for 24 h and then treated with 0.6 μmol/L MLN4924 for 48 h. Knockdown efficiency and IκBα, p-IκBα and catalase were assessed by IB analysis. **c** Downregulation of IκBα by siIκBα remarkably increased proliferation inhibition compared with MLN4924 alone. EC1 and Kyse450 cells were transfected with control or IκBα siRNA for 48 h and then treated with 0.6 μmol/L MLN4924 for 48 h. **d** Schema of the mechanism for MLN4924-induced protective autophagy in ESCC

Since Catalase serves as a classical target gene of NF-κB, we next determined whether the inhibitory effect of MLN4924 on Catalase expression and ATF3-induced autophagy is mediated by NF-κB pathway. Translocation of NF-κB to the nucleus is allowed by the phosphorylation of IκBα, resulting in its ubiquitination and degradation by CRLs complex [38]. As expected, MLN4924 treatment significantly inhibited global protein neddylation and Cullin1 neddylation, indicating the inactivation of CRL E3 ligase. Consequently, p-IκBα, a classical substrate of CRL, was dramatically accumulated upon neddylation-CRL inactivation (Fig. 5a). Moreover, IκBα knockdown markedly attenuated MLN4924-induced reduction of Catalase, accumulation of ATF3 and induction of autophagy (Fig. 5b). We further explored whether IκBα knockdown has the similar role of ATF3 knockdown-enhanced MLN4924-induced proliferation inhibition in EC1 and Kyse450 cells. As shown in Fig. 5c, IκBα knockdown also significantly increased MLN4924-induced proliferation inhibition. These findings collectively demonstrated that MLN4924 inhibited NF-κB pathway to reduce Catalase expression, which promoted the ROS generation to eventually induce ATF3 accumulation and autophagic response.

## Discussion

Esophageal cancer is the fourth largest malignant tumor of digestive system and sixth mortality rate of cancer diseases, while the novel therapeutic strategies for this deadly disease are urgently needed. Recently, protein neddylation pathway has emerged as a promising anti-ESCC target, as best supported by the discovery of overactivation of the neddylation pathway in esophageal cancer and the potent anti-ESCC effects of specific NAE inhibitor MLN4924 in preclinical trials. In the present study, we reported for the first time to our knowledge that MLN4924 treatment induced the accumulation of ATF3 and ATF3-mediated autophagy via NF-κB-Catalase-ROS pathway. Functionally, blockage of MLN4924-induced protective autophagy by ATF3 deletion sensitized ESCC cells to MLN4924-induced apoptosis. These preclinical findings indicated that NF-κB-Catalase-ROS-ATF3 axis served as a new protective autophagy mechanism in esophageal cancer upon MLN4924 treatment, which provided a rationale for combinational anti-ESCC therapy with dual inhibition of neddylation and autophagy pathways.

ATF3 is one of the crucial stress-responsive factors and acts as either a tumor suppressor or oncoprotein in cancer-type dependent manner [32, 39–42]. Generally, previous study has shown that ATF3 induced NOXA expression to promote apoptosis in chronic myelogenous leukemia (CML) cells [41]. Conversely, ATF3 could also promote proliferation of Adult T-cell leukemia (ATL) cells via mechanisms including upregulation of CDC2 and cyclin E2 [39]. Therefore, determining the role of ATF3 in ESCC development might provide potential intervention points for anti-ESCC therapy.

In this study, we found that MLN4924 treatment induced ATF3 expression to trigger autophagic response as a pro-survival signal while ATF3 silencing promoted cell apoptosis. Consistently, TCGA RNA-seq database analysis showed that ATF3 was substantially elevated in ESCC tissues when compared with the adjacent normal tissues (Fig. S1.A). Furthermore, high expression of ATF3 was negatively correlated with the prognostic survival curve in patients with esophageal cancer, suggesting that ATF3 may act as an oncogene in esophageal cancer (Fig. S1.B). Thus, our findings that MLN4924-induced ATF3 activation acts as a pro-survival event in ESCC highlights the therapeutic value of targeting ATF3 and neddylation pathway for combinational ESCC therapy.

ATF3 has been reported to regulate the expression of NF-κB target genes, such as TNF-a and IL-6 [43, 44]. Conversely, there are reports showing that NF-κB could directly induce the transcriptional activation of ATF3 [45]. In the present study, however, we showed that ATF3 was activated by MLN4924-induced ROS due to the inhibition of NF-κB-Catalase axis in ESCC cells, revealing a new mechanism by which NF-κB regulates ATF3. Further investigation of the cross-talk between ATF3 and NF-κB signaling is of great interest.

NF-κB acts as a potent pro-survival transcription factor and promotes the progression of various tumors [46–48]. However, in the present study, we found that inactivation of NF-κB pathway upon MLN4924 treatment acts as a protective role by inducing the ATF3-mediated autophagy in esophageal cancer cells. Consistently, IκBα knockdown activates NF-κB signaling to attenuate MLN4924-induced ATF3 expression and protective autophagy. Together, NF-κB pathway may have dual functions in response to therapeutic stresses, acting as a pro-death or a pro-survival signal upon treatment.

Previous studies have reported that ATF3 could induce autophagy. For example, LYN-1604, as a novel activator of ULK1, obviously up-regulated ATF3 to induce autophagy in triple negative breast cancer [49, 50]. However, the further mechanism by which ATF3 induces autophagy is largely unknown. In the present study, we showed that MLN4924 induced autophagy dependent on ATF3 accumulation. These findings establish the necessity to explore the mechanism by which ATF3 promotes autophagy in future studies.

Our study suggested the following working model (Fig. 5d). In esophageal cancer cells, MLN4924 induces the expression of ATF3 by modulating NF-κB-Catalase-ROS pathway to trigger pro-survival autophagy, whereas targeting ATF3 blocks the autophagic response upon neddylation inhibition and thus sensitizes cancer cells to MLN4924-induced apoptosis. These findings provide a potential combination strategy of dually targeting ATF3 and neddylation pathway for effective anti-ESCC therapy.

## Supplementary information

**Additional file 1.** (PPTX 156 kb)

## Data Availability

The datasets used and/or analyzed during the current study are available from the corresponding author on reasonable request.

## Abbreviations

NEDD8: neural precursor cell expressed developmentally down-regulated 8
CRL: Cullin-RING E3 ligase
ESCC: Esophageal squamous cell carcinoma
CHX: cycloheximide
CQ: chloroquine
BafA1: Bafilomycin A1
3MA: 3-methyladenine
IB: immunoblotting
