# Supplementary material for: Neddylation inhibition activates the protective autophagy through NF-κB-catalase-ATF3 Axis in human esophageal cancer cells
Source: Cell Commun Signal. 2020 May 12;18:72. doi: 10.1186/s12964-020-00576-z (PMC7218644; doi:10.1186/s12964-020-00576-z)
Supplement: Supplementary file 2 — Additional file 1. (PPTX 156 kb) [file 12964_2020_576_MOESM1_ESM.pptx]

## Slide 1
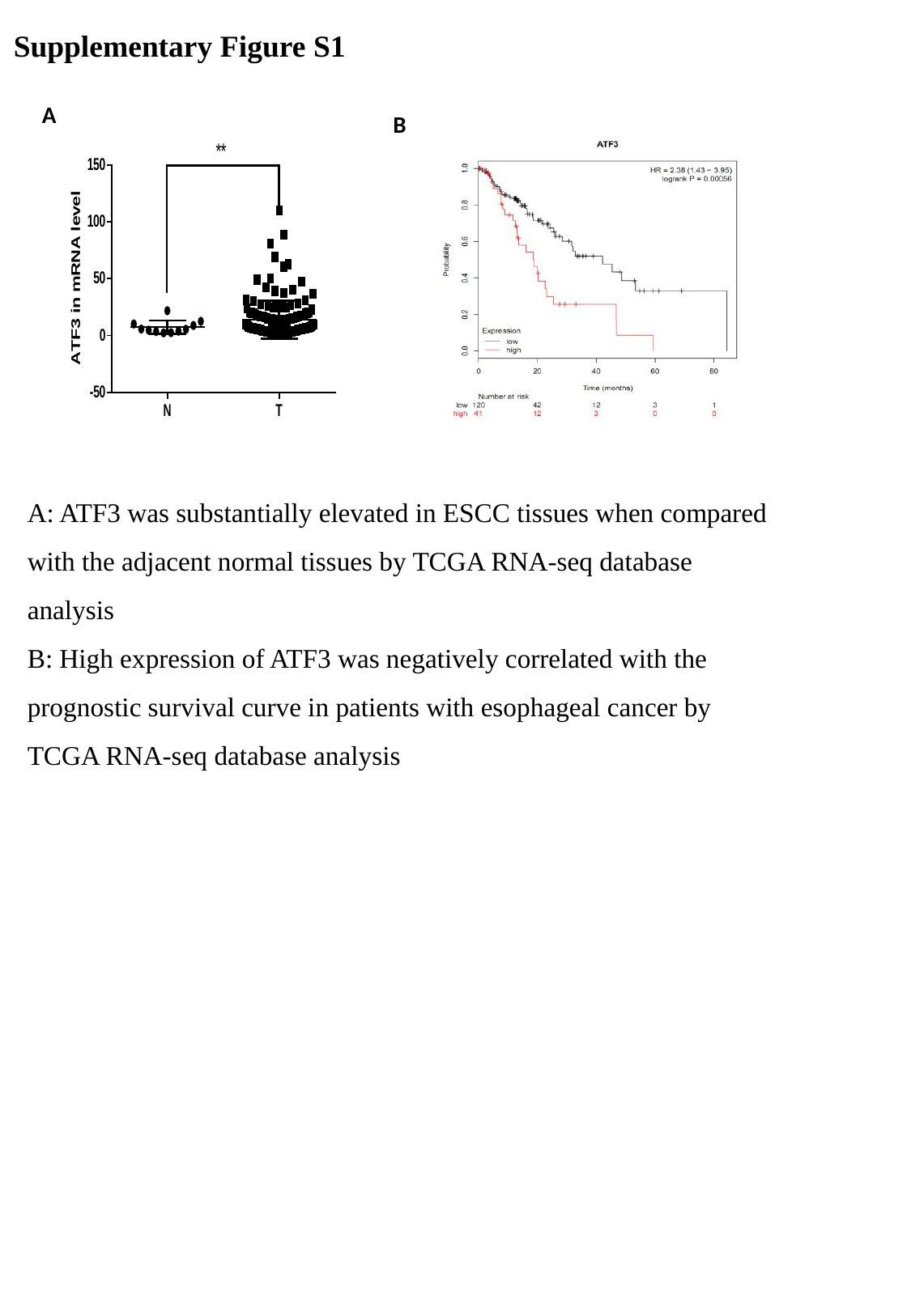

Supplementary Figure S1
A
B
A: ATF3 was substantially elevated in ESCC tissues when compared with the adjacent normal tissues by TCGA RNA-seq database analysis
B: High expression of ATF3 was negatively correlated with the prognostic survival curve in patients with esophageal cancer by TCGA RNA-seq database analysis
